# Evaluation of the efficacy of simplified nutritional instructions from physicians on dietary salt restriction for patients with type 2 diabetes mellitus consuming excessive salt: protocol for a randomized controlled trial

**DOI:** 10.1186/s13063-019-3864-8

**Published:** 2019-12-23

**Authors:** Emi Ushigome, Chikako Oyabu, Makoto Shiraishi, Nobuko Kitagawa, Aya Kitae, Keiko Iwai, Hidetaka Ushigome, Isao Yokota, Masahide Hamaguchi, Masahiro Yamazaki, Michiaki Fukui

**Affiliations:** 1 Department of Endocrinology and Metabolism, Graduate School of Medical Science, Kyoto Prefectural University of Medicine, 465 Kajii-cho, Kawaramachi-Hirokoji, Kamigyo-ku, Kyoto, 602-8566 Japan; 2 Department of Endocrinology and Metabolism, Graduate School of Medical Science, Kyoto Prefectural University of Medicine, 465, Kajii cho, Kamigyo-ku, Kyoto-city, Kyoto, 621-8585 Japan; 3 Department of Endocrinology and Metabolism, Kyoto First Red Cross Hospital, 749 Honmachi 15-chome, Higashiyama-ku, Kyoto, 605-0981 Japan; 4 Department of Organ Transplantation and General Surgery, Graduate School of Medical Science, Kyoto Prefectural University of Medicine, 465 Kajii-cho, Kawaramachi-Hirokoji, Kamigyo-ku, Kyoto, 602-8566 Japan; 5 Department of Biostatistics, Graduate School of Medicine, Hokkaido University, Kita 8, Nishi 5, Kita-ku, Sapporo, Hokkaido 060-0808 Japan

**Keywords:** Daily salt intake, Dietary salt restriction, Simplified nutritional instructions, Randomized controlled trial, Sodium, Type 2 diabetes mellitus

## Abstract

**Background:**

Hypertension is present in more than 50% of patients with type 2 diabetes mellitus. Dietary salt restriction is recommended for the management of high blood pressure. Instructions on dietary salt restriction, provided by a dietitian, have been shown to help patients reduce their salt intake. However, appointments for the dietitians in hospitals are often already fully booked, making it difficult for patients to receive instructions on the same day as the outpatient clinic visit.

**Aim:**

The aim of this trial is to test a new intervention to assess whether guidance on dietary salt restriction provided by physicians during outpatient visits is effective in reducing salt intake in patients with type 2 diabetes mellitus who have an excessive salt intake.

**Methods:**

In this unblinded randomized controlled trial (RCT), a total of 200 patients, male or female, aged between 20 and 90 years, who have type 2 diabetes mellitus and consume excessive salt will be randomly assigned to two groups: an intervention group and a control group. In addition to being given routine treatment, participants in the intervention group will be given individual guidance on restricting their dietary salt intake by a physician upon enrollment. The control group will only be given routine treatment. Participants will be followed up for 24 weeks. The primary outcome will be dietary salt intake, which will be assessed at baseline and at 8, 16, and 24 weeks. The secondary outcomes, including body weight, body mass index, hemoglobin A1c level, blood pressure, blood glucose level, serum lipid profile, and urinary albumin excretion level, will be assessed at baseline and at 8, 16, and 24 weeks.

**Discussion:**

The results of this RCT have the potential to provide a simple and novel clinical approach to reduce salt intake among patients with type 2 diabetes, making regular visits to their physician, in outpatient facilities. This protocol will contribute to the literature because it describes a practical intervention that has not been tested previously, and it may serve as guidance to other researchers interested in testing similar interventions.

**Trial registration:**

University Hospital Medical Information Network (UMIN), UMIN000028809. Registered retrospectively on 24 August 2017. http://www.umin.ac.jp.

## Introduction

According to a recent estimate, about 425 million adults around the world and an estimated 158.8 million adults in the Western Pacific Region are living with diabetes mellitus (DM) [1]. Among the International Diabetes Federation regions, the Western Pacific Region has the highest number of adults living with DM [1, 2]. Moreover, the prevalence of both type 1 and type 2 DM (T2DM) has increased significantly during recent decades [3]. T2DM, being much more common, has been the main driver for the increase in global diabetes prevalence [3]. The results of the National Health and Nutrition Survey in Japan, released in September 2017, reported that there were an estimated 20 million patients with T2DM and prediabetes, of which 10 million patients had T2DM [4].

Hypertension is present in more than 50% of patients with T2DM and contributes significantly to microvascular and macrovascular diseases in T2DM [5]. Moreover, the incidence of cardiovascular disease markedly increases when the two diseases coexist [6]. Thus, intensive treatment of high blood pressure, in addition to hyperglycemia, is important for patients with T2DM.

Epidemiological studies have shown that excessive salt intake is directly related to cardiovascular mortality and to an increase in blood pressure [7]. Many hypertension guidelines recommend salt restriction, based on studies that have demonstrated a decrease in blood pressure with a reduction in salt intake [8, 9]. We have previously reported in a single-arm trial that nutritional instructions on dietary salt restriction by a national registered dietitian provided to patients with T2DM consuming excessive salt may be beneficial in reducing dietary salt intake and home blood pressure [10]. In that study, daily salt intake was significantly reduced by 0.8 g at 2 months and 0.7 g at 6 months after the instruction. Moreover, morning systolic blood pressure was significantly reduced by 2.7 mmHg at 2 months and 5.8 mmHg at 6 months after the instruction. However, dietitians are usually too busy to see patients during their regular clinic visits. Therefore, it would have great clinical significance if this study can demonstrate that shorter and more focused nutritional instructions given to patients by their regular physicians during regular outpatient visits are effective. It might contribute to reducing the financial burden on patients and also the need for patients to have separate consultations with a dietitian.

We plan to investigate a new method of providing advice to patients with T2DM regarding dietary salt restriction for the management of hypertension: nutritional instructions will be given to patients by their regular physicians during regular outpatient visits.

### Aims of study

This randomized controlled trial (RCT) will evaluate whether simplified instructions on how to reduce salt intake, provided by a physician using standardized nutritional guidance materials, will help patients with T2DM consuming excessive salt to reduce their salt intake.

## Methods

### Study design

**Fig. 1:**
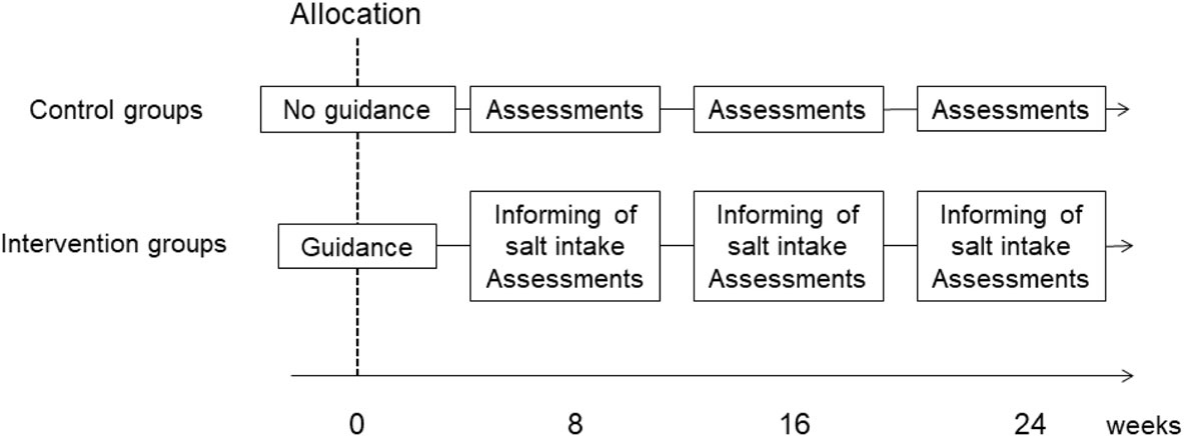
Flow chart of the study

The study will be an unblinded RCT aimed at testing whether instructions on dietary salt restriction provided by physicians during outpatient visits is effective in reducing salt intake in patients with type T2DM who have an excessive salt intake. A total of 200 patients with T2DM and excessive salt intake (150 from the Hospital of the Kyoto Prefectural University of Medicine, 50 from Kyoto First Red Cross Hospital according to the proportion of outpatients) will be randomly assigned to an intervention group or a control group, using permuted block randomization, with block sizes of ten. Spot morning urine tests will be performed, and the results will be used to estimate their salt intake. The primary outcome will be the change in daily salt intake between baseline and 24 weeks. Each participant will be enrolled for 24 weeks from randomization to the final follow-up assessment and will visit every 8 weeks (Fig. 1).

The Standard Protocol Items: Recommendations for Interventional Trials (SPIRIT) checklist is provided as Additional file 1.

### Study setting

The study will be conducted in outpatient clinics at the Hospital of the Kyoto Prefectural University of Medicine and Kyoto First Red Cross Hospital. Participants will be recruited in the outpatient clinics, and all study visits will be conducted in outpatient clinics.

### Recruitment

Participants will be recruited from April 2016 to December 2020.

### Inclusion criteria

Patients will be considered for inclusion in the study if they meet all of the following inclusion criteria: willing and able to give informed consent for participation in this study; male or female, aged between 20 and 90 years old; diagnosed with T2DM (we will apply the Japan Diabetes Society’s “Committee report on classification and diagnostic criteria of diabetes mellitus” for diagnosing diabetes [11]); and salt intake ≥ 6 g per day in patients with hypertension [12], salt intake ≥ 7 g per day in female patients without hypertension, or salt intake ≥ 8 g per day in male patients without hypertension [13]. Daily salt intake will be measured using a spot morning urine sample.

### Exclusion criteria

Patients will be excluded from the study if they have any of the following exclusion criteria: history of or features indicative of an eating disorder; secondary hypertension or malignant hypertension; pregnant, breast-feeding, or planning to become pregnant within the next 6 months; myocardial infarction, cerebrovascular accident, or hospitalization for angina pectoris within the previous 6 months; change in antihypertensive medication and/or antidiabetic medication within the previous month; cardiac failure (grade II, III, or IV according to the New York Heart Association) [14]; atrial fibrillation or severe arrhythmia; advanced renal failure (serum creatinine greater than or equal to 2.0 mg/dL or patient currently on dialysis); life-threatening condition such as a malignant tumor; intercurrent serious infection; significant psychiatric disorder or diagnosed substance abuse; serious neurological disorder, including epilepsy; unable to change their diet (currently in a facility or using home delivery); or recruiting physician considers the patient to be unsuitable for the study for any other reason.

### Study procedures

After confirming that a patient is eligible for the study, written informed consent will be obtained. Upon enrollment, the participants will be randomized to the intervention group or the control group.

#### Control group

During their baseline visit, participants allocated to the control group will be informed of their current salt intake but will not be given instructions by their physician on how to reduce their salt intake. Their physician will give them the same dietary guidance that is routinely given to patients during outpatient visits.

#### Intervention group

During the baseline visit, participants allocated to the intervention group will be informed of their current salt intake and will be given instructions by their physician on how to reduce their salt intake. This will take about 10 min. (For further details, see the Intervention section.) The intervention group will also receive routine education along with educational intervention.

All participants will be asked to attend follow-up visits 8, 16, and 24 weeks after enrollment.

### Allocation

All participants will be randomized to one of the two arms (intervention or control) in equal ratios, using permuted block randomization, with block sizes of ten. An independent researcher will draw lots to assign each participant to a group directly after obtaining the participant’s informed consent. Because of the nature of this study, it will not be possible to blind participants, physicians, or some of the researchers to the treatment allocation.

### Intervention

During the baseline visit, participants in the intervention group will be informed of their current daily salt intake and the salt intake recommendation levels (as noted in the inclusion criteria) by their regular physician, who will then give them instructions on how to reduce their salt intake, including clear recommendations for food swaps (e.g., change soy sauce to low salt soy sauce). The materials (pamphlets) used and the doctors’ instructions will be standardized. Instructions on reducing salt intake will be given using pamphlets describing “Twelve tips on reducing salt intake” (listed below); “Specific examples of amount of seasoning equivalent to 1 g of salt”; and “Concrete example of estimated amount of salt contained in food (menu for eating out, dried fish, paste products, processed food, instant food, pickles, *tsukudani*, and delicacies)”. The intervention will take about 10 min. Participants will also be informed of their current daily salt intake at the 8-week and 16-week study visits.

The 12 tips on reducing salt intake are:

1. Get used to more mild flavors.
2. Limit your intake of pickles and soup.
3. Use salt sparingly.
4. Dip, do not douse, food in salty sauces such as soy sauce.
5. Flavor food with sour-tasting ingredients.
6. Use spices liberally.
7. Use natural aromatic seasonings such as herbs and perilla.
8. Fragrant smells are your friend (grill, roast, toast, etc.).
9. Use flavorful oils such as sesame oil, olive oil, and herbal oil.
10. Limit your intake of snacks that you eat with alcohol.
11. Limit your intake of fish cakes and processed foods.
12. Do not overeat.

### Measurements and outcomes

All outcome measures will be assessed at baseline and at the 2-month, 4-month, and 6-month follow-up visits (Table 1). Data and safety monitoring will be conducted at all sites periodically during the study.

**Table 1:** Overview of enrollment and assessment schedule

	Study period				
	Enrollment	Allocation	Post-allocation		
Timepoint	–8 weeks	0	8 weeks	16 weeks	24 weeks
Enrollment:					
Eligibility screen	X				
Informed consent	X				
Allocation		X			
Interventions:					
Guidance on dietary salt restriction		X			
Informing of salt intake:					
Intervention groups		X	X	X	X
Control groups		X			
Assessments: (both groups)					
Demographics	X				
Dietary salt intake	X	X	X	X	
Medication review	X	X	X	X	
Body weight	X	X	X	X	
Blood pressure	X	X	X	X	
Hemoglobin A1c	X	X	X	X	
Fasting blood glucose	X	X	X	X	
Serum lipid profile	X	X	X	X	
Renal function	X	X	X	X	
Liver function	X	X	X	X	
Urinary sodium/potassium ratio	X	X	X	X	
Urinary albumin excretion	X	X	X	X	

#### Primary outcome

The primary outcome will be dietary salt intake and will be assessed at baseline and at 8, 16, and 24 weeks. For feasibility, daily salt intake will be measured using a spot morning urine sample and will be calculated using the following equation:

Daily salt intake (g/day) = 0.0585 × 21.98 × {urinary sodium (mEq/L)/urinary creatinine (mg/dL) × (14.89 × body weight (kg) + 16.14 × height (cm) – 2.04 × age (years) – 2244.45)}^0.392^ [15]. Spot morning urine samples will be collected using an immunoturbidimetric assay (Autokit Micro Albumin, Wako, Osaka, Japan).

#### Secondary outcomes

The secondary outcomes will include body weight, hemoglobin A1c, blood pressure, serum lipid profile (triglycerides and high-density lipoprotein cholesterol), estimated glomerular filtration rate, and urinary albumin excretion (UAE) [9]. Blood samples for biochemical measurements will be taken in the morning.

Hemoglobin A1c level, serum lipid profile, and other biochemical data will be determined using standard laboratory measurements. The UAE will be measured with an immunoturbidimetric assay. The hemoglobin A1c level will be expressed in National Glycohemoglobin Standardization Program units. Information on age, duration of diabetes, smoking status, alcohol consumption, and use of medication will be collected from each participant at baseline.

The presence of retinopathy will be assessed from chart reviews.

The presence of nephropathy will be assessed by dividing the UAE into three categories, as follows: normoalbuminuria, UAE < 30 mg/g Cr; microalbuminuria, 30–300 mg/g Cr; or macroalbuminuria, > 300 mg/g Cr.

Neuropathy will be defined based on the diagnostic criteria for diabetic polyneuropathy proposed by the Diabetic Neuropathy Study Group [16].

Macrovascular complications will be defined as the presence of previous cardiovascular disease, cerebrovascular disease, or arteriosclerosis obliterans, on the basis of clinical history or physical examination.

Changes in diabetic medication (number of drugs currently prescribed to the patient; dose of drugs; initiation of new medication during study period; initiation of injectable diabetic medication; initiation of insulin; number of medications stopped or changed during the study period) and changes in antihypertensive medication (number of drugs started and stopped during study period; dose of drugs; initiation of new medication during study period) will be recorded. We will exclude patients from the analysis who were newly prescribed or stopped thiazide diuretics and sodium glucose transporter 2 inhibitors during the study.

### Statistical methods

A mixed model will be used to analyze the factors involved in sodium intake. Factors and changes in factors that may influence sodium intake will be adjusted in multivariate models. Subgroup analyses will be conducted stratified by age (< 65 years or ≥ 65 years), sex (male or female), body mass index (< 22%, or ≥ 22%), and hemoglobin A1c levels (< 7%, or ≥ 7%) to investigate the coherency of educational effect among subgroups. The secondary outcomes will be compared using separate regression models. The differences of values between the two groups with 95% confidence interval and a *p* value < 0.05 will also be presented. We will use an intention-to-treat analysis. JMP version 11.2.0 software (SAS Institute, Cary, NC, USA) will be used as the statistical software package for the analysis. All analyses will be two-sided, and *p* values < 0.05 will be considered statistically significant. Multiple imputations will be applied if there are missing values. In a previous study, it was shown that after patients with T2DM were given instructions on reducing salt intake, their salt intake declined from 10.3 ± 1.6 g to 9.3 ± 1.9 g per day [10]. Therefore, we have based our sample size calculation on the assumption that the intervention group will show a 1-g reduction in their daily salt intake, and that the salt intake in the control group will remain unchanged. With the two-sided significance level set at 5.0% and power set at 80%, the required sample size will be 90 participants in each group. Assuming a 10% dropout rate [17], a total of 200 participants will be required for both groups combined.

### Patient and public involvement

Patients are not consulted about how to design a medical protocol, research questions, or outcome measures for an intervention relating to health outcomes. Patients are given a description of the study with their consent forms. They are also provided with results at the conclusion of the study. Control participants are offered the opportunity to be given the protocol at the completion of the study, pending positive outcomes.

## Discussion

Observational studies have indicated that most people consume an excess level of salt and that excessive salt intake may contribute to hypertension, which is a risk factor for various diseases, such as stroke, coronary heart disease, and nephropathy [7]. Therefore, salt restriction is essential for the prevention of cardiovascular disease and is recommended in many guidelines. This study is a randomized controlled trial on the effect of providing patients with simplified instructions on how to reduce salt intake from their regular physician during a regular clinic visit, using standard nutritional guidance materials. Demonstrating that shorter and more focused instructions during regular outpatient visits are effective would have great clinical significance. This protocol will contribute to the literature because it describes a practical intervention that has not been tested previously, and it may serve as guidance to other researchers interested in testing similar interventions.

Results will be reported in accordance with the Consolidated Standards of Reporting Trials (CONSORT) guidelines and will be disseminated via peer-reviewed publications and presentations at national and international conferences.

### Trial status

The trial is ongoing. The protocol version number is R000026346. Recruitment began on April 1, 2016. The approximate date when recruitment will be completed is March 31, 2020.

## Supplementary information

**Additional file 1.** SPIRIT 2013 checklist: recommended items to address in a clinical trial protocol and related documents.

## Data Availability

Not applicable.

## Abbreviations

DM: Diabetes mellitus
RCT: Randomized controlled trial
T2DM: Type 2 DM
UAE: Urinary albumin excretion
